# Inhibition of Pb*GP43* Expression May Suggest that gp43 is a Virulence Factor in *Paracoccidioides brasiliensis*

**DOI:** 10.1371/journal.pone.0068434

**Published:** 2013-07-11

**Authors:** Isaura Torres, Orville Hernandez, Diana Tamayo, Jose F. Muñoz, Natanael P. Leitão, Ana M. García, Angela Restrepo, Rosana Puccia, Juan G. McEwen

**Affiliations:** 1 Unidad de Biología Celular y Molecular, Corporación para Investigaciones Biológicas (CIB), Medellín, Colombia; 2 Facultad de Medicina, Universidad de Antioquia, Medellín, Colombia; 3 Instituto de Biología, Universidad de Antioquia, Medellín, Colombia; 4 Facultad de Ciencias de la Salud, Institución Universitaria Colegio Mayor de Antioquia, Medellín, Colombia; 5 Departamento de Microbiologia, Imunologia e Parasitologia, Escola Paulista de Medicina - Universidade Federal de São Paulo, São Paulo, Brazil; Louisiana State University, United States of America

## Abstract

Glycoprotein gp43 is an immunodominant diagnostic antigen for paracoccidioidomycosis caused by *Paracoccidioides brasiliensis*. It is abundantly secreted in isolates such as Pb339. It is structurally related to beta-1,3-exoglucanases, however inactive. Its function in fungal biology is unknown, but it elicits humoral, innate and protective cellular immune responses; it binds to extracellular matrix-associated proteins. In this study we applied an antisense RNA (aRNA) technology and *Agrobacterium tumefaciens*-mediated transformation to generate mitotically stable Pb*GP43* mutants (Pb*GP43* aRNA) derived from wild type Pb339 to study its role in *P. brasiliensis* biology and during infection. Control PbEV was transformed with empty vector. Growth curve, cell vitality and morphology of Pb*GP43* aRNA mutants were indistinguishable from those of controls. Pb*GP43* expression was reduced 80–85% in mutants 1 and 2, as determined by real time PCR, correlating with a massive decrease in gp43 expression. This was shown by immunoblotting of culture supernatants revealed with anti-gp43 mouse monoclonal and rabbit polyclonal antibodies, and also by affinity-ligand assays of extracellular molecules with laminin and fibronectin. *In vitro*, there was significantly increased TNF-α production and reduced yeast recovery when Pb*GP43* aRNA1 was exposed to IFN-γ-stimulated macrophages, suggesting reduced binding/uptake and/or increased killing. *In vivo*, fungal burden in lungs of BALB/c mice infected with silenced mutant was negligible and associated with decreased lung ΙΛ−10 and IL-6. Therefore, our results correlated low gp43 expression with lower pathogenicity in mice, but that will be definitely proven when Pb*GP43* knockouts become available. This is the first study of gp43 using genetically modified *P. brasiliensis*.

## Introduction

*Paracoccidioides brasiliensis* is a thermally dimorphic Ascomycota that causes paracoccidioidomycosis (PCM), a prevalent systemic mycosis endemic in Latin America, where up to ten million people can be infected [1]. It is generally accepted that the infection is acquired through inhalation of conidia produced by the mycelial phase of the fungus, which at body temperatures grows as multibudding yeast [2]. Gp43 is the main *P. brasiliensis* diagnostic antigen and the best characterized fungal molecule so far [3], [4], [5], [6]. It is a secreted glycoprotein that contains only one high mannose oligosaccharide chain bearing a single β-galactofuranosyl terminal residue [7]. Intracellularly, gp43 is stored inside large cytoplasmic vacuoles and lomasomes, while secretion seems to take place at specific regions of the cell wall, where antigen aggregates can be visualized in immunogold-labeled yeasts [8]. The Pb*GP43* gene encodes a 416-amino-acid long protein bearing a leader sequence of 35 residues [9]. Two exons are separated by a 78-bp intron, while a marked polymorphism in exon 2 has been fundamental to define phylogenetic groups within the species [10], [11]. Although the translated protein is structurally related to cellulases, with 58% similarity with an exo-beta-1,3-glucanase from *Candida albicans*, enzymatic activity *in vitro* was absent [9] (Cisalpino et al., 1996).

Serological diagnosis and prognosis of PCM are generally performed using simple double immunodiffusion tests. Whole *P. brasiliensis* extracellular preparations are used where gp43 is the antigenic component responsible for 85–100% positivity with sera from patients with both acute and chronic PCM [12], [13], [14], [15], [3]. False-negative reactions have been found only in patients with intense pulmonary infection and immune depression [12], [16]. Therefore, most *P. brasiliensis* isolates express gp43 during infection. However, *in vitro* some are high producers, like the isolate Pb339, while others can barely express it [17]. Culture conditions and fungal status clearly contribute to the level of antigen expression *in vitro*, but little is known about transcriptional control [18], [19].

Cell wall gp43 is at least partially responsible for yeast cell adhesion to components of the extracellular matrix. *P. brasiliensis* adhesion to Vero cells was inhibited by anti-gp43 polyclonal serum [20]. Purified gp43 was able to bind to laminin-1 from mouse sarcoma at a K_d_ of 3.7 nM [21], while laminin and fibronectin internal peptides competed for gp43 adhesion to the corresponding proteins [22]. These results suggested that cell wall gp43 might help mediate cell-cell interaction in lung alveoli by binding to laminin and fibronectin. Penetration of the fungus in the tissues, however, would depend on the action of proteases degrading extracellular matrix-associated proteins (ECM). In this sense, the extracellular thiol-dependent serine protease that preferentially cleaves ECM might act as a virulence factor when associated with gp43 [23], [24]. The high mannose moiety appended to gp43 is partially responsible for fungal binding and uptake by macrophages via the mannose receptor [25]. As far as gp43 presentation to the immune system is concerned, dendritic and B cells are also involved, thus resulting in activation of, respectively, Th1- and Th2-related cytokines [26]. Presentation by macrophages results in the stimulus of both types of cytokines by lymphocytes collected from infected mice.

Besides being highly antigenic for antibody production, gp43 elicits T-CD4^+^ protective response mediated by intense IFN-γ production in mice immunized with the protein or its gene [27], [28]. An internal sequence of 15 amino acids (P10) does not elicit antibody production, thus avoiding interference of a Th-2 unwanted response [28]. The P10 peptide or its gene can efficiently be used both as vaccine and immunotherapy, especially if associated with anti-fungal agents [29], [30], [31]. Conversely to its protective properties, exposure to gp43 decreased the phagocytic index of bone marrow-derived macrophages to zymozan [32]. This effect was mimicked by two internal peptides that also inhibited nitric oxide and stimulated hydrogen peroxide production, and evoked anti-inflammatory effects in the initial stages of footpad swelling stimulated by *P. brasiliensis* yeasts [32], [33].

In this work, we have addressed the role of gp43 in *P. brasiliensis* yeast cells during interaction with macrophages and a mouse model of infection. We generated a *P. brasiliensis* strain with reduced Pb*GP43* expression using antisense RNA and *A. tumefaciens*-mediated transformation (ATMT) [34], [35], [36]. Our data further confirms that gp43 seems to play an important role during host-pathogen interaction as the low expression of wild-type gp43 protein levels led to reduced fungal burden in mice lungs.

## Materials and Methods

### Ethics Statement

This study was carried out as recommended by the Brazilian college of animal experimentation (COBEA). The experimental protocols and procedures were reviewed and approved by the Ethical Committee on Animal Experimentation of Federal University of Sao Paulo, UNIFESP (Permit number: 0366/07).

### Strains and Culture Media

We used *P. brasiliensis* Pb339, which is known to produce high amounts of extracellular antigens, especially gp43 [13], [37], [38]. Yeast cells maintenance and growth curves were performed in BHI media supplemented with 1% glucose (Beckton Dickinson and Company, Sparks, MD) at 36°C with aeration in a mechanical shaker and were routinely collected during early exponential phase (72–96 h).

Selection of *P. brasiliensis* transformants was performed in BHI solid media, supplemented with hygromycin B (100 µg/ml) after a 15-day incubation period at 36°C. *Escherichia coli* DH5α grown in Luria Bertani (LB) at 36°C supplemented with appropriate antibiotics was used for cloning and propagation of plasmids [39]. *Agrobacterium tumefaciens* strain LBA1100 [40] was used to carry the binary vectors used in this study; recombinant *A. tumefaciens* was maintained in LB medium supplemented with kanamicin (100 µg/ml). Morphological transitions from mycelium to yeast, and germination from yeast to mycelium were carried out in BHI agar medium at 36°C or 20°C, respectively.

### Strategy to Obtain PbGP43 aRNA *P. brasiliensis* Strains

We followed the anti-sense strategy and *A. tumefaciens*-mediated transformation [35] to obtain *P. brasiliensis* mutants with silenced Pb*GP43*. Based on the genomic sequence from Pb339 (PbWt) originally reported by Cisalpino et al., 1996 [9] (GenBank accession No. AY005437) we amplified three anti-sense oligonucleotides designated as AS1 (base pairs 133–291 of Pb*GP43*, exon 1), AS2 (base pairs 54–177 of Pb*GP43*, exon 2) and AS3 (base pairs 323–484 of Pb*GP43*, exon 2). They were individually inserted into the pCR35 plasmid under the control of *H. capsulatum* calcium-binding protein gene (*CBP-1*) promoter [41], and propagated in *E. coli* DH5α. Each *CBP-1* promotor-AS cassette was subcloned into pUR5750, which was used as a parental binary vector to harbour the transfer DNA (T-DNA) with antisense sequences. As a control, we transformed Pb339 yeasts with an empty vector (PbEV), as previously described [35], [36]. To evaluate the phenotypic stability of the isolated *Agrobacterium*-mediated transformants they were randomly selected and subcultured in liquid BHI containing 100 µg/ml hygromycin B for three consecutive times for 5 days at 36°C and then evaluated again in solid medium with three biological replicates before their use in others assays.

### Molecular Detection of the Hygromycin Resistance Gene (HPH)

Genomic DNA from PbWt, PbEV, and Pb*GP43* aRNA (anti-sense mutants) yeast cells were isolated according to the glass beads protocol described by Van Burik [42]. In order to confirm the presence of the hygromycin B resistance cassette, PCR analysis was carried out to detect an *HPH* 1000-bp amplification product using primers *hphF* (5′-AACTCACCGCGACGTCTGTCGA-3′) and *hphR* (5′-CTACACAGCCATCGGTCCAGA-3′). PCR amplification included 30 cycles of 1 min at 94°C, for denaturing, 1 min at 68°C for annealing, and 1.5 min at 72°C for extension. The reaction products were analyzed in 1% agarose gel and visualized with ethidium bromide under UV light.

### Total RNA Extraction and Real-time RT-PCR Analysis

Total RNA was obtained from PbWt, PbEV, and Pb*GP43* aRNA yeast cells using the TRIzol® reagent according to the manufacturer’s instructions (Invitrogen, Carlsbad, CA, USA). Total RNA was treated with DNase I (Invitrogen, Carlsbad, CA, USA) and tested for chromosomal DNA contamination using conventional PCR for β-tubulin gene [43]. cDNA was synthesized using 1 µg of total RNA and SuperScript III reverse transcriptase according to the manufacturer’s instructions (Invitrogen, Carlsbad, CA, USA).

Real-time PCR (RT-qPCR) was performed using Maxima® SYBR Green/Fluorescein qPCR Master Mix, according to the manufacturer’s instructions (Fermentas Maryland, USA). The CFX96 Real-Time PCR Detection System (Bio-Rad, Headquarters Hercules, California, USA) was used to estimate Pb*GP43* expression; β-tubulin was selected as house keeping gene [43]. Melting curve analysis was performed after the amplification phase to eliminate the possibility of nonspecific amplification or primer-dimer formation. Fold changes in mRNA expression were calculated using the 2^ΔΔCT^ formula, where ΔΔCT is the difference between target and β-tubulin genes [44]. Each experiment was carried out in triplicates and the expression level was measured three times.

We evaluated the expression of interleukin 6 (IL-6), IL-10, IL12p40, and tumor necrosis factor alpha (TNF-α) in a cell line (MH-S), which corresponds to mouse alveolar macrophages transformed with SV40, obtained from the European Collection of Cell Cultures (ECACC No 95090612). The cells were activated or not with IFN-γ during interaction with *P. brasiliensis* strains at different time points (1, 3, 6, 12, 24 and 48 h). The expression levels were measured in triplicates in three individual assays. RNA samples were obtained from MH-S cells after each interaction time point using the TRIzol reagent according to the manufacturer’s instructions (Invitrogen). The ubiquitin gene (*UBI*) was used as housekeeping gene control [36]. The primer name and sequences used in RT-qPCR, are listed in table 1.

**Table 1:** Oligonucleotide primer pairs used in RT-qPCR.

Primer name	Primer Sequence 5′-3′
Pb*GP43*RT-F	TCGGCAGAGATGCTAAAAGGC
Pb*GP43*RT-R	TTCTGACATGGTTTAACCCCG
β-tubulin-F	GTGGACCAGGTGATCGATGT
β-tubulin-R	ACCCTGGAGGCAGTCACA
IL-6-F	ACACATGTTCTCTGGGAAATCGT
IL-6-R	AAGTGCATCATCGTTGTTCATACA
IL-10-F	TTTCAATTCCCTGGGGGAGAA
IL-10-R	GCTCCACTGCCTTGCTCTTATT
IL-12p40-F	CAAATTACTCCGGACGGTTCA
IL-12p40-R	AGAGACGCCATTCCACATGTC
TNF alpha-F	GCCACCACGCTCTTCTGTCT
TNF alpha-R	TGAGGGTCTGGGCCATAGAAC
Ubiquitin-F	TGGCTATTAATTATTCGGTCTGCAT
Ubiquitin-R	GCAAGTGGCTAGAGTGCAGAGTAA

### Growth Curve and Vitality Assays

Growth curves were performed in BHI broth (100 mL) inoculated with 48 h cultures (1×10^8^ washed cells). Cellular density was measured in duplicates at 3, 6, 12, 24, 48, 72, 120 and 144 h of growth using a spectrophotometer.

Vitality was evaluated using the protocol reported by Hernandez et al. [36], which corresponds to the ability of yeast cells to metabolize glucose upon late activation of a cell membrane proton pump and subsequent acidification of the medium due to H^+^ release. The acidification power in presence of glucose is a good predictive test of yeast vitality [45] because it allowed observing the physiological capabilities and metabolic functions of the cell. For that, PbWt, PbEV, and Pb*GP43* aRNA yeast cells were cultivated in BHI liquid medium and collected at 48 h of growth, washed twice with sterile water pH 7.0, and suspended in a final volume of 8 ml of water (pH 7.0). Two milliliters of this suspension were added to a beaker containing 38 ml of water and when the pH became stable (pH 5.5 to 6), 10 ml of 20% glucose were added. The pH was recorded every three min for 1 h in order to evaluate the increase in supernatant H^+^ levels. PbWt yeast cells treated with 16 µg/ml of amphotericin B (Fungizone, Bristol-Myers Squibb Pharmaceuticals, England) during 4 hours was used as negative control of the assay. The assays were performed in triplicates.

### Evaluation of gp43 in Culture Supernatants

*P. brasiliensis* yeast cells (mutant and controls) were recovered from 5-day-old slants of modified yeast extract peptone dextrose (YPDmod: 0.5% yeast extract; 1.0% casein peptone; 0.5% glucose, pH 6.3), and inoculated into 10 ml of YPDmod. This pre-inoculum was incubated for 5 days at 36°C in a rotatory shaker at 120 rpm. Log-phase cells, collected by gravity, were inoculated in 100 ml of YPDmod (1×10^8^ cells/ml) and incubated at 36°C. Aliquots were taken at 3, 6, 9 and 12 days of growth for analysis of culture supernatants and cell extracts. The presence of gp43 in the samples was monitored by sodium dodecyl sulfate polyacrylamide gel electrophoresis (SDS-PAGE) followed by silver staining and Western blots.

### SDS-PAGE and Western Blot

SDS-polyacrylamide gel electrophoresis (SDS-PAGE) was performed as described by Laemmli [46]. Culture supernatants from controls and mutant Pb339 (60 µl and 150 µl) were heat-concentrated in SDS-PAGE buffer under denaturing conditions and resolved in 10% gels. The gel contents were transferred to 0.45-mm nitrocellulose membranes [47] and blotted proteins were assayed. For Western immunoblotting, all incubations steps were carried out with shaking. The membranes were quenched overnight at 4°C with 5% non-fat milk in PBS and incubated for 3 h in phosphate-saline buffer (PBS) containing 10 µg/ml of anti-gp43 MAb17c monoclonal antibodies [4], or with anti-gp43 rabbit immune serum (1∶10,000) [38]. After three washes (10 min/each) in PBS/0.1% Tween 20 (PBST), the nitrocellulose sheets were incubated for 1 h with either goat anti-mouse IgG (1∶1,000) conjugated with peroxidase (Sigma) or anti-rabbit IgG-peroxidase (1∶1,000; Invitrogen) in 5% nonfat milk in PBS. The membranes were washed three times in PBST, followed by a last wash with PBS. The immunocomplexes were developed by a chemiluminescent method (Immobilon™ Western Chemiluminescent HRP Substrate).

### Ligand-affinity Assays

To determine the capacity of supernatant components to bind to ECM proteins, we used the protocol reported by Donofrio et al. [48], with modifications. Culture supernatants from control and mutant *P. brasiliensis* were resolved by SDS-PAGE and transferred to nitrocellulose membranes [47]. Blotted proteins were assayed for laminin and fibronectin affinity as follows: the membranes were incubated with 1% bovine serum albumin (BSA) in PBS, pH 7.4, for 4 h at room temperature and then for 90 min in PBS-T-BSA (PBS, 0,05% Tween 20, 5% non fat milk) containing 30 µg/ml of either laminin (derived from Engelbreth-Holm-Swarm murine sarcoma from Sigma) or human fibronectin (Sigma). Membranes were washed three times in PBS-T, 10 min each, and then incubated for 1 h under agitation with rabbit anti-mouse laminin or rabbit anti-human fibronectin antibodies (both from Sigma, 1∶100) in PBS-T-BSA under continuous agitation. The blots were washed in PBS-T and incubated with peroxidase-labeled goat anti-rabbit immunoglobulin at 1∶1,000 in PBS-T-BSA at room temperature. The membranes were washed and the reactions were developed using a chemiluminescent method (Immobilon™ Western Chemiluminescent HRP Substrate).

### Antifungal Activity of MH-S Activated with IFN-γ

A cell line (MH-S), which corresponds to mouse alveolar macrophages transformed with SV40, was obtained from the European Collection of Cell Cultures (ECACC No 95090612). IFN-γ-activated alveolar macrophages were grown in RPMI 1640 medium supplemented with 2 mM glutamine (Invitrogen, Carlsbad, CA, USA), 0.05 mM 2- mercaptoethanol (Sigma Aldrich, USA), and 10% fetal bovine serum (Invitrogen, Carlsbad, CA, USA). For the assays, we used confluent monolayers obtained by adding 4×10^5^ cells per well to 24-well tissue culture plates (Nunc, Kamstrup, Denmark) incubated for 24 h at 36°C with 5% CO_2_ prior to evaluating interaction with PbWt, PbEV and Pb*GP43* aRNA1 yeast cells. Macrophage monolayers were activated for 18 h by adding recombinant IFN-γ to a final concentration of 10 µg/ml (BD Pharmingen™) and incubated overnight at 37°C under 5% CO_2._ Non-activated macrophages were used as internal control of the experiment. The MH-S cells (activated and non activated with IFN-γ) were challenged with 8×10^4^ yeasts/well diluted in 250 µl of complete RPMI medium and incubated at 37°C for 1, 3, 6, 12, 24 and 48 h. After interaction, survival of *P. brasiliensis* yeast cells was evaluated using the CFU (colony forming units) method. At each time point, cultures were rinsed with RPMI to remove free yeast cells (supernatant), and then distilled water was added to lyse IFN-γ activated and non-activated macrophages and then PbWt, PbEV, and Pb*GP43* aRNA1 cells were removed. Dilutions of both suspensions (supernatant and intracellular/adhered fungi) were plated onto BHI plates supplemented with 0.5% glucose, 4% horse serum, and EDTA 300 mM and incubated at 37°C for 5 to 8 days as previously described [49]; the results obtained from the intracellular/adhered fungi suspension were compared with the number of yeast cells recovered in the supernatant, in order to control the number the yeast cells added to each well. Percentage of viable cells was expressed as the number of CFUs obtained from each experimental well (*P. brasiliensis* yeast cells with IFN-γ-activated alveolar macrophages) divided by the number of CFUs in the controls (*P. brasiliensis* yeast cells growing in RPMI-serum without macrophages interaction). All experiments were performed in triplicates.

### In vivo Assays

BALB/c mice were bred at the CEDEME (Centro de Desenvolvimento de Modelos Animais) of Federal University of São Paulo, under specific-pathogen-free conditions and were kept with food and water *ad libitum*. All animals were handled according to both national and international guidelines for animal research, and all possible steps were taken to minimize animal suffering in these experiments. Groups of 10 BALB/c male mice (10-week old) were inoculated intratracheally with 1×10^6^ viable yeast cells/50 µl PBS/animal of each fungal sample (PbWT, PbEV, and Pb*GP43* aRNA1) from YPDmod slants [50]. Mice were anesthetized intraperitoneally with 80 mg/kg of ketamine and 10 mg/kg of xylazine. After approximately 10 min, the necks were hyper extended, and the tracheas were exposed at the level of the thyroid and injected with the fungal cells. The incision was sutured with 5-0 silk. The number of viable microorganism in lungs of infected mice was determined by CFU counts. After 50 days of infection the lungs were removed, weighed, homogenized, and washed three times in PBS by centrifugation; the final pellets were suspended in 1 ml of PBS. Aliquots (100 µl) of each homogenate were plated onto BHI agar that contained 5% (vol/vol) BFS (bovine fetal serum, Invitrogen) and 10% *P. brasiliensis* culture filtrate that constituted the source of growth promoting factors [51]. Plates were incubated at 36°C and colonies were counted daily until no increase in counts was observed. The number (log_10_) of viable *P. brasiliensis* per gram of tissue was expressed as the means±standard errors.

### Cytokine Detection

Lungs were homogenized in 2 ml of PBS in the presence of protease inhibitors: benzamidine HCl (4 mM), EDTA disodium salt (1 mM), N-ethylmaleimide (1 mM) and pepstatin (1.5 mM) (Sigma, St Louis, MO). The supernatants were assayed for IL-6, IL-10, IL-12, TNF-α and IFN-γ using ELISA kits (BD OpTeia, San Diego, CA). The detection limits of the assays were as follows: 7.8 pg/ml for IL-6, 31.3 pg/ml for IFN- γ, 62.5 pg/ml for IL-12, 15.6 pg/ml for TNF-α and IL-10, as previously determined by the manufacturer. Assays were performed with a pool of lung homogenates from all the mice in each group.

### Statistics

Data are reported as mean ± standard error of the mean (SEM) and all assays were repeated at least three times. All statistical analyses were performed using SPSS statistics 17.0 program with ANOVA. A *p* value less than or equal to 0.05 was considered statistically significant.

## Results

### Inhibition of PbGP43 Expression does not Affect Morphology, Vitality or Fungal Growth

**Figure 1:**
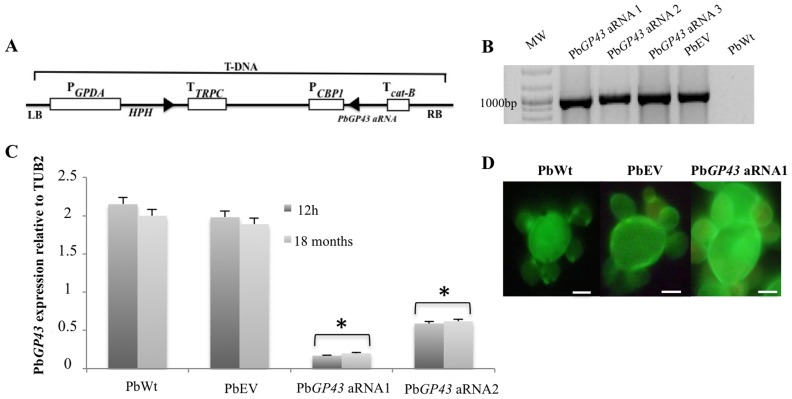
Inhibition of Pb*GP43* expression using an aRNA plasmid and *A. tumefaciens*-mediated transformation. (**A**) Pb*GP43* anti-sense cassette. Three anti-sense oligonucleotides were produced (AS1, AS2, AS3) based on Pb339 (PbWt) genomic sequence, as detailed in Materials and Methods, and cloned under the control of the *H. capsulatum CBP-1* promoter (P). The constructs were sub-cloned into the T-DNA region of the binary vector pUR5750 harboring the *E. coli HPH* resistance gene driven by the GPDA promoter from *A. nidulans* and bearing transciptional terminators (T) from *cat-B* and *TRPC*; LB, left border; RB right border. (**B**) PCR fragments amplified with *HPH* specific primers to yield a 1,000-bp internal fragment. We used as template DNA from Pb*GP43* aRNA (AS3, clones 1, 2, 3), PbWt and PbEV. Strains were tested before and after exhaustive subculturing in selective and non-selective medium with similar results. MW, DNA molecular marker. (**C**) Pb*GP43* expression levels in PbWt, PbEV, and Pb*GP43* aRNA (1 and 2) after 1 month and over 18 months of subculture. Gene expression levels obtained by RT-qPCR were normalized with the internal control *TUB2*; *, *p*<0.05 when compared with PbWt and PbEV. (**D**) Inhibition of Pb*GP43* expression did not alter the morphology of Pb*GP43* aRNA1 that showed round multibudding yeasts similar to control strains PbWt and PbEV. Cellular morphology of exponentially growing yeast cells was visualized by fluorescence microscopy using calcofluor white staining. White bars correspond to 5 µm.

Using anti-sense (aRNA) technology and *A. tumefaciens*-mediated transformation (ATMT) we generated Pb*GP43* aRNA strains to further study the role of gp43 in *P. brasiliensis*. We tested cassettes containing three different aRNA oligonucleotides (AS1, AS2, and AS3), which corresponded to homologous sequences in both Pb*GP43* exons (Fig. 1A). Several hygromycin-resistant (HygR) transformants were selected from each tested aRNA oligonucleotide to evaluate mitotic stability by successive subculturing in selective and non-selective medium. Mitotic stability (after 64 subcultures) was confirmed up to 18 months after ATMT. We also confirmed integration of the hygromicin cassette in the genomic DNA of different HygR transformants (Fig. 1B).

**Figure 2:**
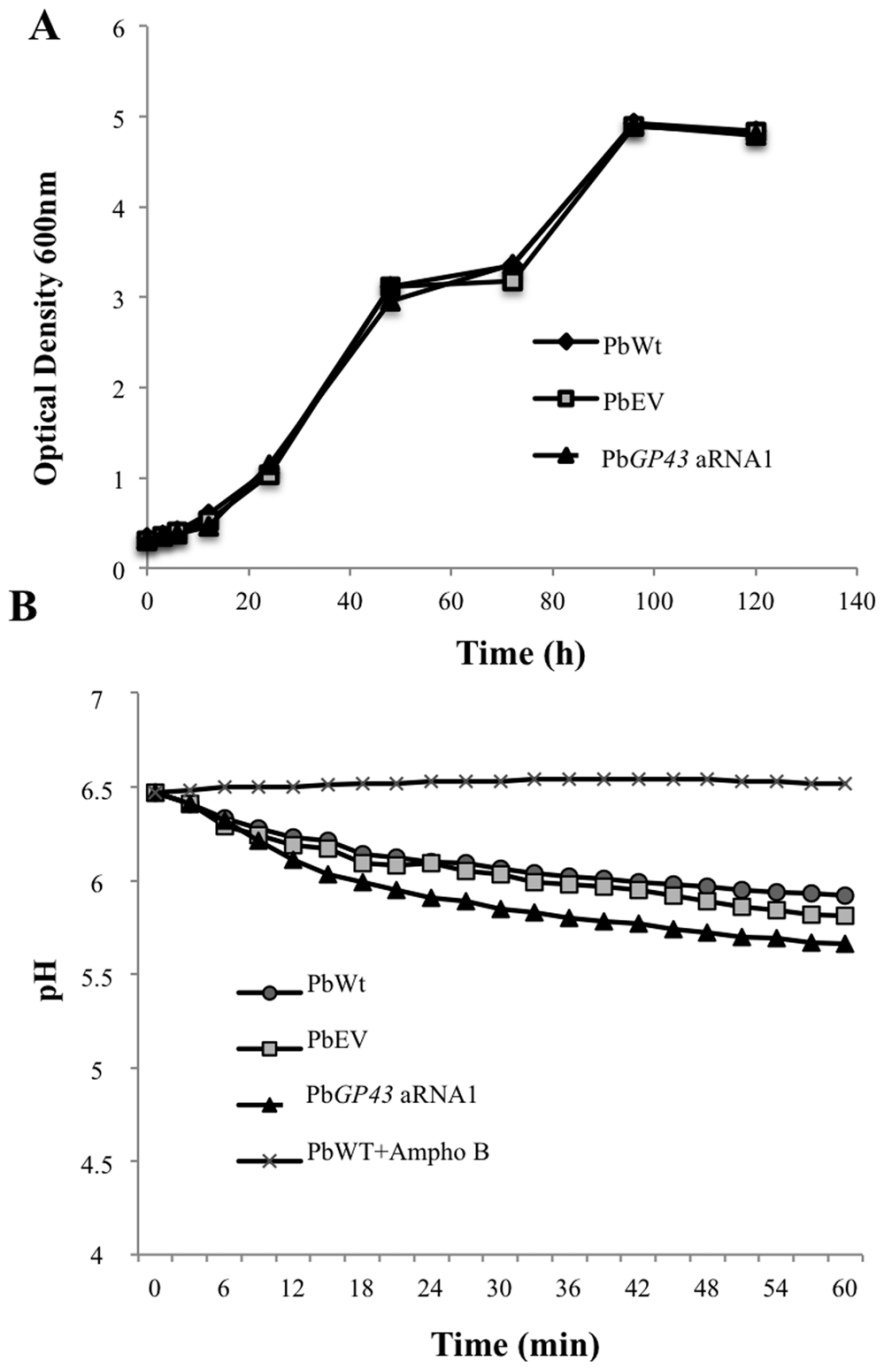
Down regulation of Pb*GP43* does not affect viability or vitality of transformed *P. brasiliensis* yeast cells. Yeast-phase *P. brasiliensis* PbWt, PbEV and Pb*GP43* aRNA1 were evaluated by (**A**) culture turbidity was determined at Optical Density 600 nm and (**B**) culture medium pH during *in vitro* growth in BHI. PbWt yeast cells treated with 16 µg/ml of amphotericin B during 4 hours was used as negative control of the assay. The assays were performed in triplicate.

To further confirm silencing of Pb*GP43* we selected HygR transformants 1 and 2 (Fig. 1B) and evaluated mRNA transcript levels by RT-qPCR (Fig. 1C). A reduction in Pb*GP43* gene expression as high as 85% was confirmed even after 18 months of successive subcultures of Pb*GP43* aRNA1, which was then used in the assays shown in Fig. 1D and further experiments. Under light and fluorescence microscopy, no morphological changes were observed in the Pb*GP43* aRNA1 mutant in comparison with PbWt and PbEV (Fig. 1D). Also, reduction in Pb*GP43* expression did not alter yeast cell growth during batch culture (Fig. 2A) nor cell vitality (Fig. 2B).

### Inhibition of PbGP43 Gene Expression Correlates with Decreased gp43 Production

**Figure 3:**
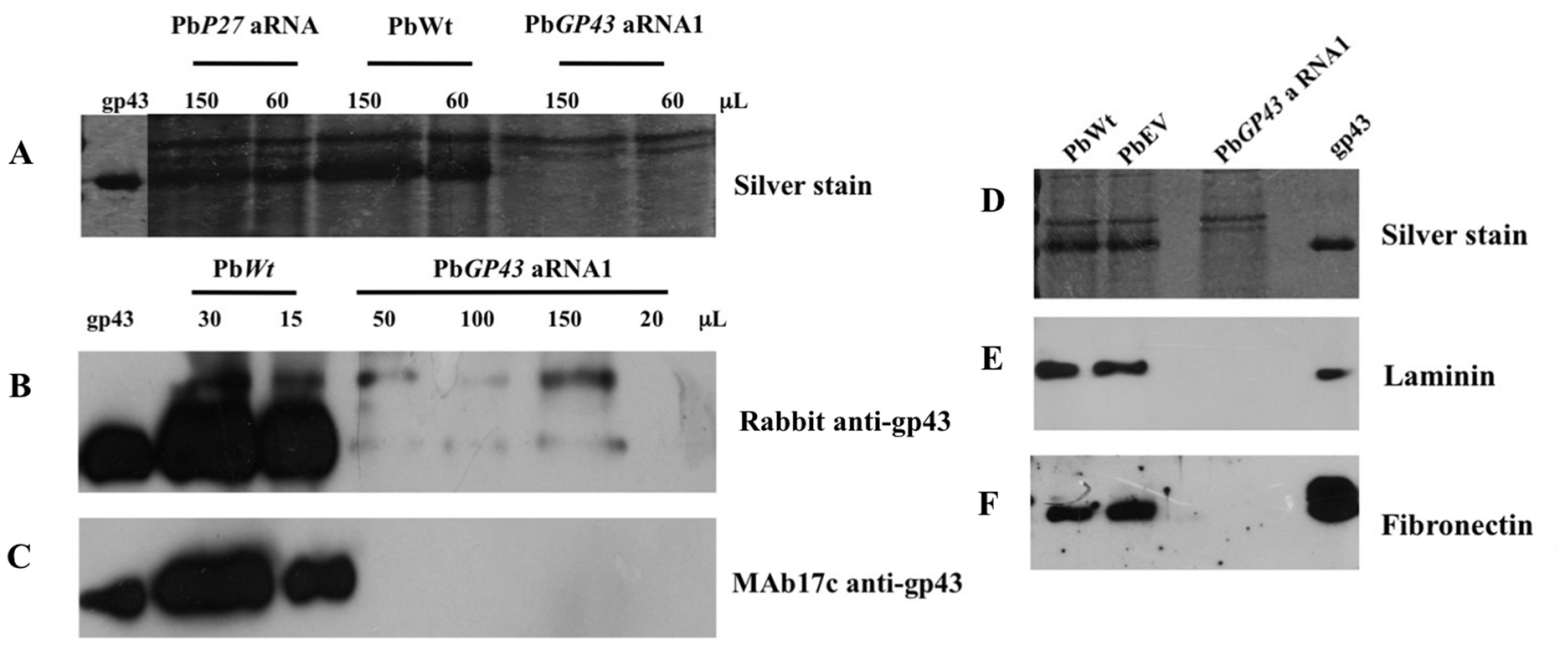
Pb*GP43* aRNA mutants showed reduced levels of secreted gp43. (**A**) Silver stained poliacrylamide gel of culture supernatant (day 12) from PbWt and mutant Pb*GP43* aRNA1 at the indicated volumes. P27 low-expression strain (Pb*P27* aRNA) was used as Pb339 recombinant control. (**B** and **C**) Immunoblots of culture supernatants tested with anti-gp43 polyclonal and monoclonal antibodies. Culture supernatant volumes are indicated. Purified native gp43 (50 ng) was used as control. (**D**) Silver stained SDS-PAGE gel of *P. brasiliensis* culture supernatants from strains PbWt, PbEV and Pb*GP43* aRNA1. (**E** and **F**) Ligand affinity assays with the samples analysed in (D), (E) laminin, and (F) fibronectin (30 µg/ml). Purified native gp43 (50 ng) was used as control.

Prior to analysing the role of gp43 during host-pathogen interaction, we confirmed that aRNA silencing of Pb*GP43* indeed led to reduction in gp43 protein expression. Therefore, we performed both SDS-PAGE gels under reducing conditions (Fig. 3A) and immunoblotting with polyclonal rabbit anti-gp43 antiserum [38] (Fig. 3B) and anti-gp43 MAb17c (Fig. 3C), which recognizes all gp43 isoforms [14]. We initially analysed gp43 expression in PbWt culture supernatants during growth (not shown) and selected day 12 samples considering that they represented the peak of extracellular gp43 expression (Fig. 3A). Extracellular gp43 was predominant among supernatant components in control strains PbWt, PbEV and Pb*P27* aRNA (used as recombinant control, while it was negligible in the Pb*GP43* silenced strain (Fig. 3A, 3D). This observation was confirmed in immunoblots with rabbit polyclonal anti-gp43 antiserum (Fig. 3B) and MAb17c (Fig. 3C), suggesting that the Pb*GP43* aRNA1 strain expressed only trace levels of the protein. This was corroborated by the lack of reactivity with gp43 when intracellular and SDS-soluble debris from the tested cultures were analysed (data not shown).

We also performed affinity-ligand assays with laminin and fibronectin of culture supernatants as an alternative test to show the absence of gp43. Note in Fig. 3D that gp43 was only detected extracellularly in PbWt and PbEV. Accordingly, laminin and fibronectin binding to gp43 was detected only in these samples (Fig. 3E and 3F). This experiment clearly shows reduced gp43 protein levels in Pb*GP43* aRNA strains when compared to the control strains. Other supernatant ligands were not detected within the range of protein concentration used in the assay.

### gp43 Plays a Role during Interaction of Yeast Cells with Macrophages

**Figure 4:**
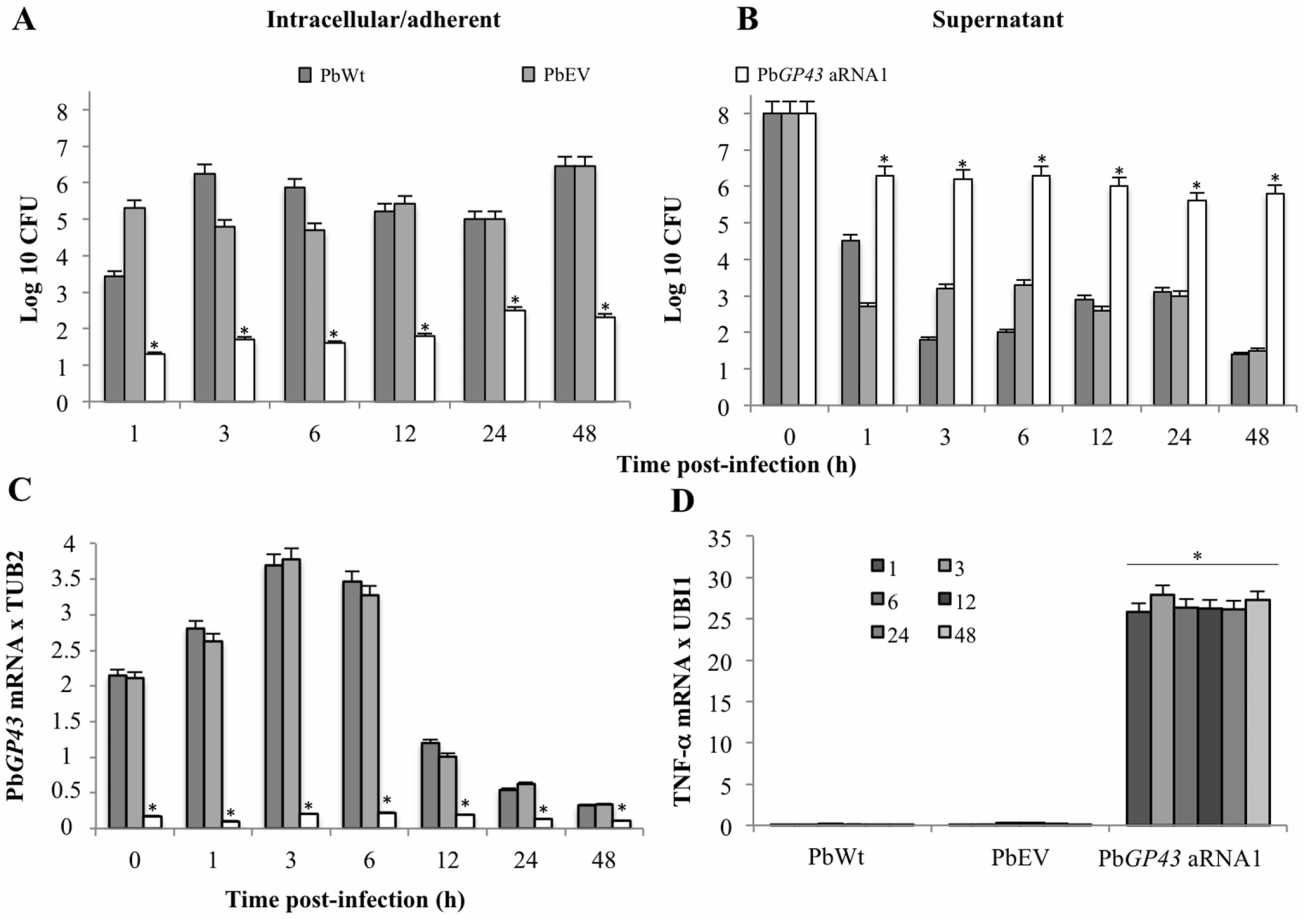
Down regulation of Pb*GP43* expression correlated with decreased recovery of intracellular/adhered yeasts from murine MH-S macrophages. (**A**) Recovered CFU from lysed IFN-γ activated macrophages; (**B**) Recovered CFU from supernatants of *P. brasiliensis*-infected culture macrophages; (**C**) gene expression levels of Pb*GP43* (relative to *TUB2*) during macrophage interaction assays with PbWt, PbEV, and Pb*GP43* aRNA1 yeasts. The periods evaluated were 0, 1, 3, 6, 12, 24 and 48 hours after interaction (*, *p*<0.05 when compared with PbWt); (**D**) TNF-α gene expression (relative to *UBI1*). The assays were performed in triplicates. Error bars indicate standard deviation.

To preliminarily elucidate the role of gp43 during host-pathogen interaction, we challenged IFN-γ-activated macrophages with PbWt, PbEV, and Pb*GP43* aRNA1 yeast cells. Percentage of Pb*GP43* aRNA1 viable yeast cells recovered from the intracellular/adhered fungi was significantly lower than that of the other strains at all tested time points (Fig. 4A). Fig 4B shows CFU recovered from the supernatant, which was a control of the CFU recovered from *P. brasiliensis* and macrophages interaction and of the quantity of yeast cells added in each experimental well. Note that the amount of yeasts recovered from the supernatant of Pb*GP43* aRNA1 infected cells was higher than from controls, suggesting impaired adherence.

Additionally, we evaluated Pb*GP43* gene expression levels during interaction of yeast cells with activated macrophages. Pb*GP43* expression was consistently reduced in the Pb*GP43* aRNA1 strain (Fig. 4C), both in the absence (not shown) and presence of activated macrophages. Both PbWt and PbEV showed increased expression of Pb*GP43* particularly during the first hours of the assay, independently of the absence (not shown) or presence of activated macrophages. The reasons for gene downregulation after 6 hours of culture are presently unknown.

We also analysed expression of TNF-α during yeast-macrophage interaction (Fig. 4D). Absence of wild-type levels of gp43 drastically increased expression of TNF-α when compared to PbWt and PbEV. We also tested the expression of interleukins (IL) IL-6, IL-10, IL12p40; however they could not be detected throughout the assays in any of the tested conditions (data not shown).

### gp43 is Important for *P. brasiliensis* Survival in a Mouse Model of Infection

**Figure 5:**
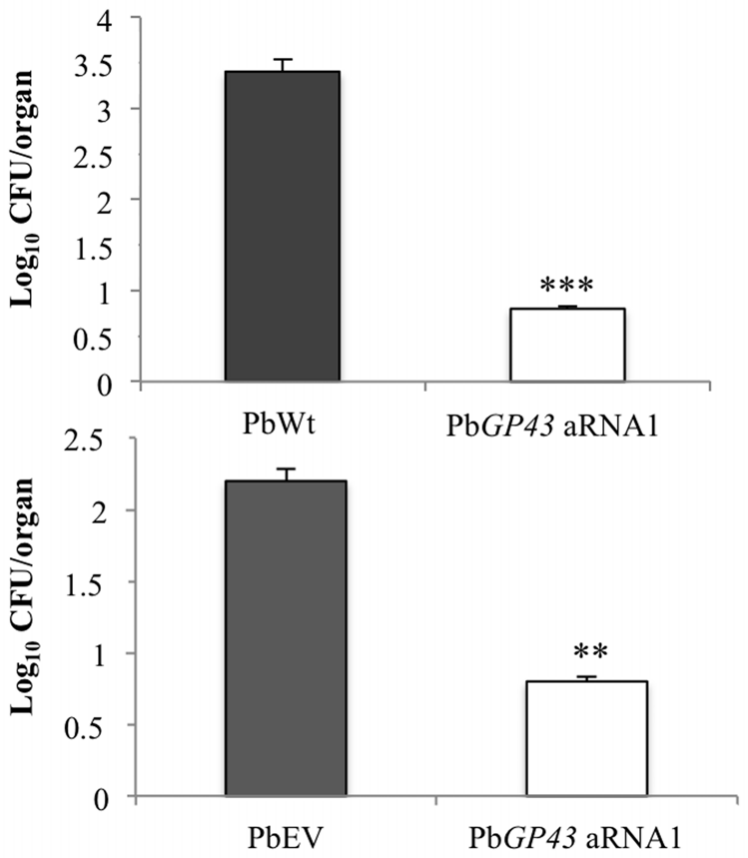
Down regulation of Pb*GP43* correlated with reduced fungal burden in lungs of infected BALB/c mice. Graphs represent CFU (expressed as log_10_ values) recovered from lung homogenates 50 days after intratracheal infection with 1×10^6^ viable yeasts of PbWt, PbEV or Pb*GP43* aRNA1. Eight mice per fungal strain were used and error bars indicate standard deviations. *P* values (**p<0.0071, ***p<0.0002) represent significant differences in comparison with the PbWt or PbEV.

In order to evaluate the role of gp43 during *P. brasiliensis* infection, we inoculated BALB/c mice intratracheally with PbWt, PbEV or Pb*GP43* aRNA1 yeast cells and measured lung fungal burden at 7 weeks post-infection (Fig. 5). The fungal burden in lungs of mice infected with the Pb*GP43* aRNA1 strain was significantly lower than that recovered from lungs infected with the PbWt or PbEV (by log_10_ of 3.4 and 2.2, respectively). Random CFUs were selected to confirm mitotic stability, Pb*GP43* aRNA gene silencing, and reduced protein expression in yeast cells.

IL-6, IL-10, IL-12 TNF-α and IFN-γ were estimated in the lungs of infected BALB/c mice. The results (Table 2) show that IL-10 and IL-6 were statistically decreased in mice lungs infected with a gp43 low-expression mutant. The response of TNF-α and IFN-γ are appparently richer, but not statistically significant.

**Table 2:** Cytokine levels in lung homogenates of BALB/c mice infected i.t. with *P. brasiliensis* PbWT, PbEV and Pb*GP43* aRNA1 yeast cells after 7 weeks of infection.

Cytokinespg/ml	TNF-α	IFN-γ	IL-12	IL-10	IL-6
PbWt	54.6±12.1	17.8±2.1	2319.0±438	330.6±12.5	133.5±8.9
PbEV	48.5±24.8	14.8±4.4	1934.8±262	283.3±9.8	119.8±7.6
Pb*GP43*aRNA1	87.5±19.1	21.3±6.2	1730.9±250.4	177.0±13.2*	98.3±7.01*

* Statistically significant difference (P<0.05) relative to mice infected with PbWT or PbEV.

## Discussion

Several studies have attempted to elucidate the function of gp43 in *P. brasiliensis* biology and pathogenesis [52], [20], [22], [3], [21]. These studies were restricted to the use of anti-gp43 antibodies, which have explored indirectly the gp43 function, however without manipulating its expression. This is the first study using a *P. brasiliensis* strain with reduced gp43 transcript and protein levels to elucidate the role of this glycoprotein during host-pathogen interaction. In all assays, a *P. brasiliensis* Pb*GP43* aRNA1 silenced strain with confirmed reduction in gene and protein expression and mitotic stability (64 subcultures) was used, as well as both wild PbWt and vector PbEV controls. Silenced strains showed similar vitality and yeast growth rates as controls. In addition, transition to the mycelial morphotype was comparable to the wild type pattern when the growth temperature was shifted to 20°C (data not shown). Therefore, apparently a decrease in Pb*GP43* gene expression does not affect important biological aspects of the parasitic yeast growth phase of the fungus in vitro.

Decrease in Pb*GP43* gene expression levels in *P. brasiliensis* Pb339 yeast cells correlated with decreased recovery of intracellular/adhered yeasts from IFN-γ-activated macrophages and from lungs of BALB/c mice infected with the Pb*GP43* aRNA1 strain. Thus, there was a reduction in the fungal burden in the lungs of mice infected with this strain, probably due to a more efficient immune response mediated by reduced levels of IL-10 and IL-6 and apparent increase in IFN-γ and TNF-α. Furthermore, in our experimental conditions, gp43 was a prevalent extracellular component to bind to ECM-associated proteins in *P. brasiliensis*. It is important to note that we have not detected in the mutant strain the presence of gp43 in intracellular fractions or debris containing cell wall (data not shown) either. Altogether, these data suggest that the lack of cell wall gp43 may have impaired binding to host cell proteins, yeast cells phagocytosis, and consequently the action of proteases responsible for fungal tissue penetration [24]. Mendes-Gianinni and colleagues observed a relationship between virulence and adherence to ECM by comparing various *P. brasiliensis* isolates that showed different degrees of virulence *in vivo* [22]. These authors also showed that Pb18 re-isolated from animals showed higher capacity to adhere to ECM components than those highly sub-cultured.

On the other hand, gp43 high-mannose oligosaccharide chains are partially be responsible for adherence and uptake of yeast cells by mouse peritoneal macrophages [25]. Our present results suggest that yeast cells from Pb*GP43* aRNA1 were either more effectively destroyed than the controls by IFN-γ-stimulated macrophages, or less bound/phagocyted by macrophages. However, increased TNF-α transcript level were only observed in macrophages in contact with the Pb*GP43* aRNA1 strain, which suggests a possible gp43 immunomodulatory effect that would be in concert with enhanced phagocytosis and/or killing. That would be in agreement with the idea that gp43 bears internal sequences that might inhibit nitrogen mediators involved in the microbicidal activity of macrophages, as reported by Konno et al. [32], [33]. To support this hypothesis it has been demonstrated that patients with chronic forms of PCM produce lower levels of IFN-γ and TNF-α, suggesting that these cytokines are important to control the development of the disease [53]. Albeit considering that trace amounts of gp43 are still expressed in the knockdown mutant, however, it is possible that this would be enough to confer protective effect by the P10 peptide [28]. On the other hand, it is clear that in the absence of gp43 other protective antigens and minor ligands could be acting. Other protective antigens eliciting an effective cellular immune response have been studied in *P. brasiliensis*, such as the 27-kDa recombinant protein (rPb27) [54]. Immunization with rPb27 in the presence of *Propionibacterium acnes* and aluminium hydroxide prior to intravenous infection by the orbital plexus with virulent *P. brasiliensis* (Pb18) was able to protect BALB/c mice against infection. Recently other recombinant protein, rPb40, was used associated with fluconazole and shown to reduce the fungal burden in lungs of BALB/c mice [55], [56]. Vaccination with recombinant Hsp60 also protected BALB/c mice against a lethal challenge with *P. brasiliensis* yeasts by stimulating cellular immune response rich in IL-12 and IFN-γ.

In summary, our results using a Pb339-derived isolate with silenced Pb*GP43* gene expression correlated gp43 with TNF-α immunomodulation of macrophages *in vitro*, and with lower pathogenicity in mice probably due to increased IFN-γ/TNF-α and decreased IL-10/IL-6 production in lungs. Additionally, decreased gp43 expression to trace levels did not affect vitality, growth curve, morphology and dimorphic capacity of fungal cells. Altogether, our results may suggest that gp43 is a virulence factor in the sense that although it did not have an effect on the survival and growth of Pb339 *in vitro*, it affected survival in a mammalian host [57]. Since total silencing of gene expression was not possible to obtain, our assumption will only be confirmed when Pb*GP43* knockouts become available.
